# Impact of inherent biases built into proteomic techniques: Proximity labeling and affinity capture compared

**DOI:** 10.1016/j.jbc.2022.102726

**Published:** 2022-11-19

**Authors:** Claudia Maria do Nascimento Moreira, Cristina D. Kelemen, Samson O. Obado, Farnaz Zahedifard, Ning Zhang, Fabiola B. Holetz, Laura Gauglitz, Bruno Dallagiovanna, Mark C. Field, Susanne Kramer, Martin Zoltner

**Affiliations:** 1 Department of Cell and Developmental Biology, Biocenter, University of, Würzburg, Würzburg, Germany; 2 Carlos Chagas Institute (ICC), FIOCRUZ/PR, Curitiba, Brazil; 3 Department of Parasitology, Faculty of Science, Charles University in Prague, Biocev, Vestec, Czech Republic; 4 The Rockefeller University, Laboratory of Cellular and Structural Biology, New York, New York, USA; 5 School of Life Sciences, University of Dundee, Dundee, United Kingdom; 6 Institute of Parasitology, Biology Centre, Czech Academy of Sciences, České Budějovice, Czech Republic

**Keywords:** affinity capture, proteome, cryomilling, BioID, interactome, BiP, binding immunoglobulin protein, BSA, bovine serum albumin, DAPI, 4′,6-diamidino-2-phenylindole, dc, dichroic, em, emission, ER, endoplasmic reticulum, ex, excitation, eYFP, enhanced YFP, FDR, false discovery rate, HA, hemagglutinin, LFQ, label-free quantification, MS, mass spectrometry, NPC, nuclear pore complex, PABP, poly(A)-binding protein, PPI, protein–protein interaction, RBP, RNA-binding protein, RT, room temperature, SigA, significant class A, SigB, significant class B, SigC, significant class C

## Abstract

The characterization of protein–protein interactions (PPIs) is of high value for understanding protein function. Two strategies are popular for identification of PPIs direct from the cellular environment: affinity capture (pulldown) isolates the protein of interest with an immobilized matrix that specifically captures the target and potential partners, whereas in BioID, genetic fusion of biotin ligase facilitates proximity biotinylation, and labeled proteins are isolated with streptavidin. Whilst both methods provide valuable insights, they can reveal distinct PPIs, but the basis for these differences is less obvious. Here, we compare both methods using four different trypanosome proteins as baits: poly(A)-binding proteins PABP1 and PABP2, mRNA export receptor MEX67, and the nucleoporin NUP158. With BioID, we found that the population of candidate interacting proteins decreases with more confined bait protein localization, but the candidate population is less variable with affinity capture. BioID returned more likely false positives, in particular for proteins with less confined localization, and identified low molecular weight proteins less efficiently. Surprisingly, BioID for MEX67 identified exclusively proteins lining the inner channel of the nuclear pore complex (NPC), consistent with the function of MEX67, whereas the entire NPC was isolated by pulldown. Similarly, for NUP158, BioID returned surprisingly few PPIs within NPC outer rings that were by contrast detected with pulldown but instead returned a larger cohort of nuclear proteins. These rather significant differences highlight a clear issue with reliance on a single method to identify PPIs and suggest that BioID and affinity capture are complementary rather than alternative approaches.

Most proteins function as part of multisubunit complexes, and identification of protein–protein interactions (PPIs) is valuable for understanding function. Identification of PPIs can uncover a wide range of interactions, which include direct, indirect, static, or dynamic binding. Furthermore, proteins can moonlight and engage in multiple different specific complexes, whereas complex composition can change in a temporal manner and/or a spatial manner.

Presently, there are two common methods used to identify PPIs in a cellular context: affinity capture (colloquially pulldown) (1) and proximity labeling (2). While evidence indicates that both methods provide valuable insights, there are considerable differences between them, both in technical requirements and consequently the interactome revealed. Each method has its adherents and while it would be fallacious to view one or the other approach as superior, it is unclear what choice of method implies in terms of the types of PPIs identified and hence critical assessment of a given dataset.

Affinity capture requires cell rupture with the lysate or extract then exposed to a solid phase with a specific affinity to the protein of interest or bait. The solid phase is commonly coupled to an antibody against the bait, or alternatively, the bait is genetically fused to a tag and purified using a solid phase with affinity to the tag. The bait along with copurified interaction partners is then eluted, and this eluate is analyzed. A major disadvantage here is that interactions are determined from the cell extract and not the true cellular environment, and thus, some interactions may be lost, whereas spurious and nonphysiological interactions may occur as a result of membrane breakage and decompartmentalization of protein complexes. Screening of extraction conditions including detergent solubilization, pH, ionic strength, and other parameters is therefore necessary. A variation on this approach is cryomilling, which avoids the use of detergents during lysis, but still disrupts cellular organization (3, 4). In general, affinity capture requires a lot of optimization for each individual bait, and results tend to be more variable between experiments and/or labs because of minor differences in cell harvesting, lysis, and buffer conditions.

In proximity labeling, some of the pitfalls of affinity isolation are avoided by capturing interactions *in vivo.* Here, the bait is fused to an enzyme that converts a substrate into a reactive radical that is covalently linked to nearby proteins. Modified proteins are purified frequently under extremely stringent conditions. The most commonly used method is BioID and variants. The bait is coupled to BirA∗, a mutant form of biotin ligase from *Escherichia coli*. Wildtype BirA converts biotin to biotinol-5′-AMP, which is retained by BirA until it is transferred to acetyl-CoA carboxylase. A mutant version, BirA∗, is modified to release biotinol-5′-AMP, causing biotinylation of lysine residues of proteins nearby (5, 6, 7). Additional BioID variants have been developed, one of which is TurboID, which exhibits greater biotinylation efficiency (8). TurboID biotinylates within minutes after addition of exogenous biotin, in contrast to other BirA∗ variants (8), but also has some biotinylation activity in the absence of exogenous biotin, leading to an increased labeling radius (9).

Given the distinct underlying principles of these two methods, we have carried out a direct comparison between them, employing four different proteins from trypanosome mRNA metabolism that encompass a variation of location and positional constraints. For affinity capture, we used data previously published by us (10, 11) and repeated the experiment for one target, nucleoporin NUP158, for optimal comparability. Cell lysis was performed by cryomilling, where rapid freezing and mechanical cell disruption at 77 K preserves PPIs and has been successful for isolating nuclear pore complexes (NPCs) and many other complexes from many organisms (3, 12, 13, 14). All proteins were expressed as GFP/YFP fusions from the endogenous locus and captured with an anti-GFP single-chain nanobody (10, 11). For BioID, we expressed the same proteins from their endogenous loci fused to the biotin ligase TurboID and used steady-state biotinylation, facilitated by biotin in the culture medium.

We found surprisingly little concordance between PPIs identified from affinity capture and BioID. With their less-confined cytoplasmic localizations, BioID is error prone for poly(A)-binding proteins (PABPs), with many false identifications. In contrast, for the identification of MEX67 interactors, BioID was more adequate and exclusively identified FG-repeat nucleoporins, NPC components that line its inner surface, rather than the entire NPC, which was isolated by the affinity method. Likewise, NUP158 affinity capture identified most part of the NPC cellular structure including distant proteins that are indirectly but stably associated with the bait, which are outside the BioID labeling radius. Whilst affinity capture delivers a snapshot of stable PPIs at the time of lysis, proximity labeling records a history of protein interactions occurring during the duration of labeling, which can bias against detection of a stable “core” complex in favor of dynamic associations. Altogether, our data indicate that the utility of each method is context dependent and hence should be viewed as complementary rather than as alternatives.

## Results and discussion

### Establishing TurboID in trypanosomes

We selected four trypanosome proteins as bait to establish BioID with TurboID biotin ligase fusion. PABP1 and PABP2 are cytoplasmic and by light microscopy appear unconfined within the cytoplasm under standard culture conditions (10, 15). The nuclear export receptor MEX67 shuttles between the nucleus and the cytoplasm with predominant localization at NPCs (16, 17). The NPC protein NUP158 (ortholog to Nup145 in *Saccharomyces cerevisiae* and Nup98/96 in *Homo sapiens*) is localized to the outer rings of the NPC (3). We selected these bait proteins for the following reasons: (i) we anticipate differential levels of nonspecific background biotinylation dependent on how rigidly a protein is confined, and including a range of protein localizations (not confined, semiconfined, and confined) allows this to be considered and (ii) prior work means we have validated cell lines and in some cases mass spectrometry (MS) data (10, 11).

**Figure 1:**
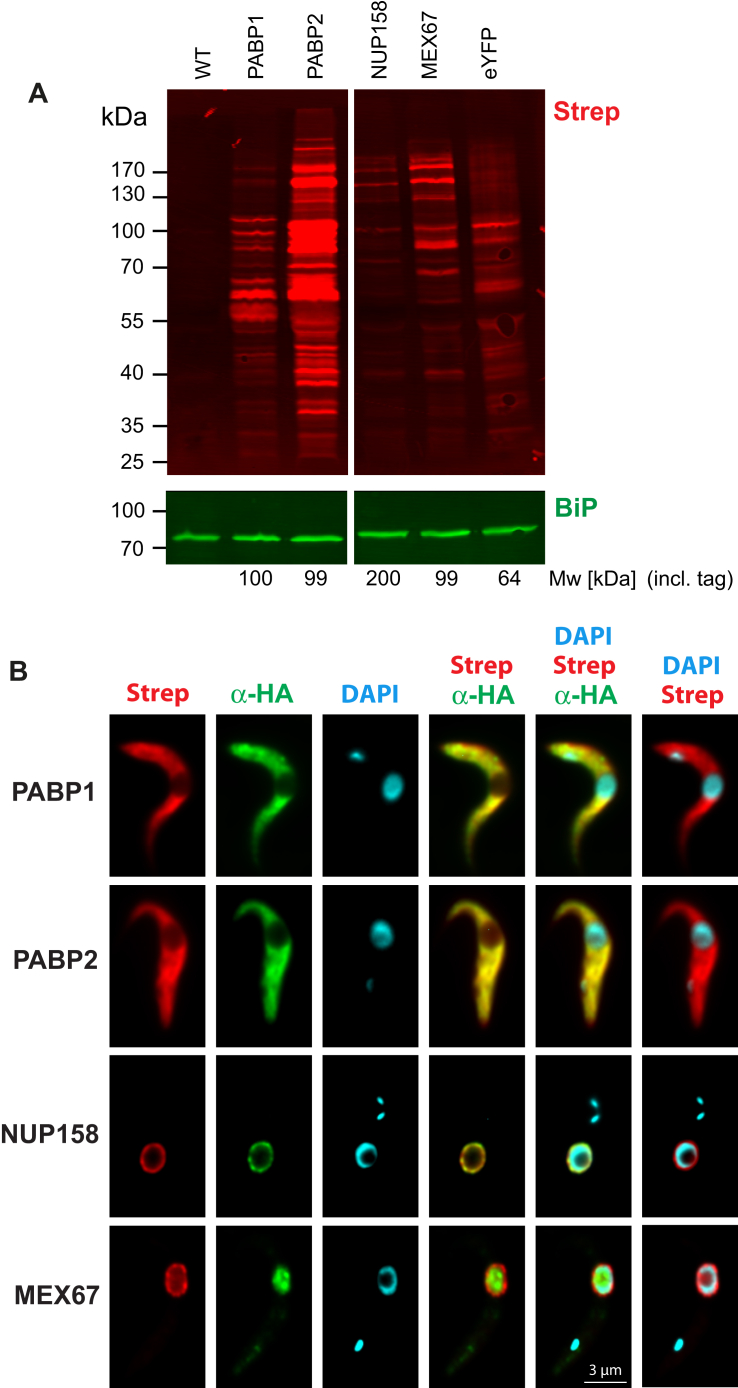
**Biotinylation in the different BioID cell lines.***A*, Western blot probed with fluorescently labeled streptavidin to detect biotinylated proteins in the control cell lines (eYFP = TurboID fused to eYFP) or cell lines expressing the respective bait proteins (PABP1, PABP2, MEX67, and NUP158) fused to TurboID. The expected molecular weight of all bait proteins fused to TurboID is indicated. *B*, localization of bait proteins and biotin labeling. Cells were probed with anti-HA antibody and IRDye 800CW streptavidin to detect biotinylated proteins, and then DAPI stained analyzed by fluorescence microscopy. DAPI, 4′,6-diamidino-2-phenylindole; eYFP, enhanced YFP; HA, hemagglutinin; PABP, poly(A)-binding protein.

All four proteins were expressed from their endogenous loci fused C-terminally to TurboID and hemagglutinin (HA), replicating the tagging strategy used for cryomill affinity capture, except that in that case, fusion was to enhanced YFP (eYFP). We used two control cell lines: unmodified parental cells and cells expressing eYFP fused to TurboID and HA (using an inducible expression system). All experiments were done in procyclic form cells, the *Trypanosoma brucei* life-cycle stage in the insect host. Generated lines were subjected to Western blotting to detect biotinylated proteins by a streptavidin probe (Fig. 1*A*). Almost no biotinylated proteins were detected in parental cells, whereas the tagged cell lines, including the eYFP control, had many biotinylated proteins. There were major differences in the number of biotinylated proteins obtained for each bait and also in the identity of the detected proteins, indicating specificity. Notably, PABP2-TurboID resulted in a significantly higher number of biotinylated proteins than PABP1-TurboID in both life cycle stages, consistent with previous findings that PABP2 interacts with a significantly bigger cohort of proteins compared with PABP1 (10). In addition, we confirmed correct localization of the TurboID bait proteins by anti-HA immunofluorescence and, in parallel, traced biotinylation *via* a fluorescent streptavidin probe (Fig. 1*B*). For the cytoplasmic PABPs, we observed cytoplasmic localization of the two probes and for NUP158 colocalization at distinct structures of the nuclear envelope. MEX67 TurboID yielded a similar streptavidin labeling pattern as NUP158 with some additional weak signal from the nucleus, but immunofluorescence signals were restricted to nucleoplasm and nucleolus. The absence of immunofluorescence from the nuclear envelope can be explained by the phase-separated environment created by the FG-type nucleoporins, which presumably prevents antibody binding in this region of the pore but not streptavidin binding. Altogether, the observed labeling pattern indicates high spatial labeling selectivity of our TurboID approach. Hence, we purified biotinylated proteins by streptavidin affinity for each cell line in triplicate and analyzed by LC–MS/MS.

### Defining confidence intervals

**Figure 2:**
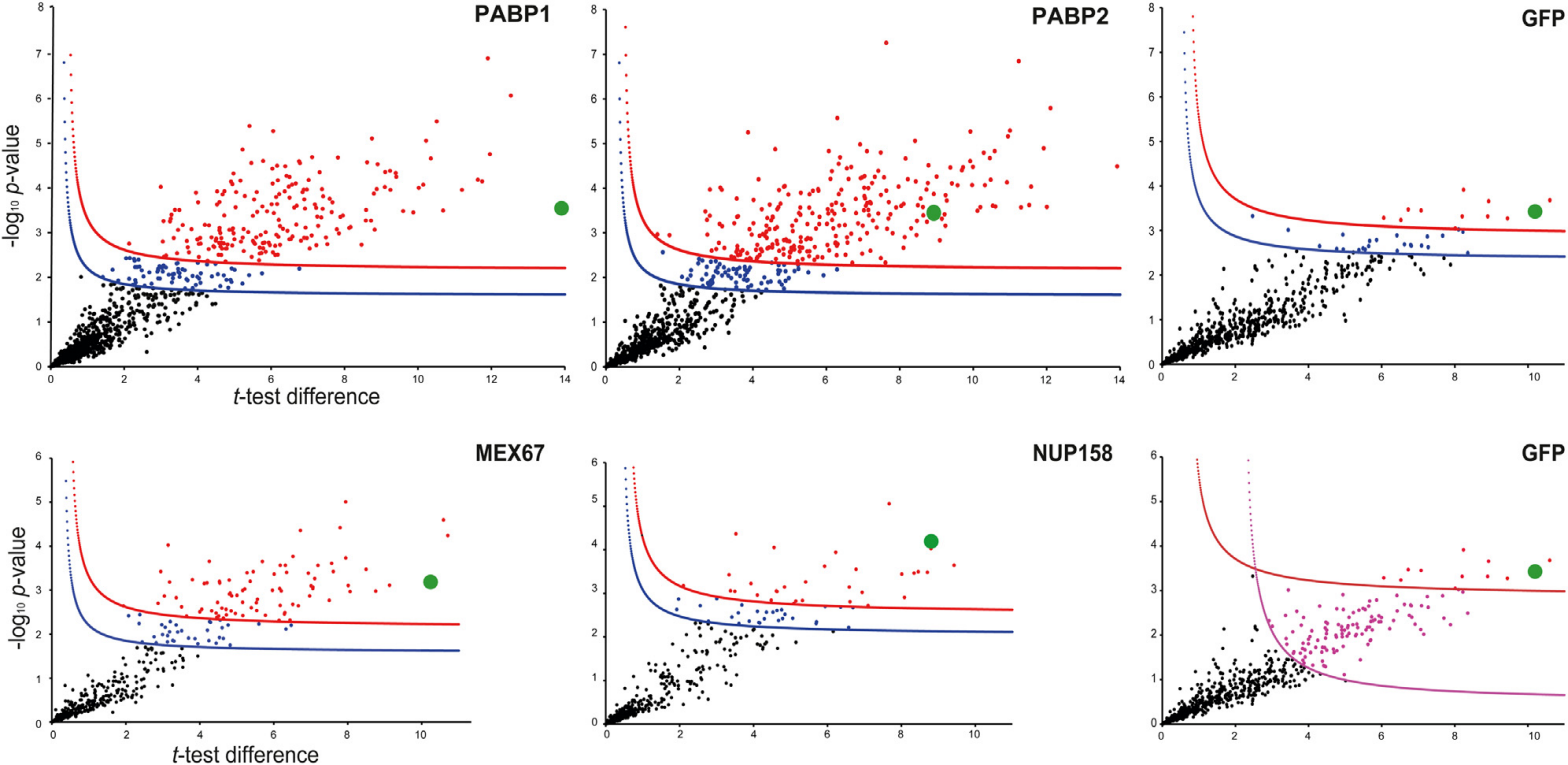
**Hawaii plot for the statistical analysis of BioID experiments.** Hawaii plot (multiple volcano plots) of LFQ results of the BioID experiments for PABP1, PABP2, MEX67, NUP158, and the GFP-control. All samples were prepared in triplicate. To generate the volcano plots, the −log_10_*p* value was plotted *versus* the *t* test difference (difference between means), comparing each respective bait experiment to the *wt* control. Potential interactors were classified according to their position in the plot, applying cutoff curves for “significant class A” (SigA; drawn in *red*; FDR = 0.01, s0 = 0.1) and “significant class B” (SigB; drawn in *blue*; FDR = 0.05, s0 = 0.1), respectively, and, for the GFP control “significant class C” (SigC; drawn in *pink*; FDR = 0.05, s0 = 2). Bait proteins are indicated by a *green dot*. FDR, false discovery rate; LFQ, label-free quantification; PABP, poly(A)-binding protein.

Proteins enriched in the BioID samples were grouped into confidence intervals (significant classes; SigA, SigB, and SigC) defined by cutoff curves in volcano plots (Fig. 2) and based on statistical analysis in Perseus (Max Planck Institute of Biochemistry, Martinsried) (18) (for details, see the Experimental procedures section). All BioID samples were compared with parental cells to identify proteins naturally biotinylated as well as proteins that nonspecifically bind to the affinity matrix. A second control, cells expressing a GFP-TurboID fusion, served to identify proteins biotinylated in a nonspecific manner.

**Figure 3:**
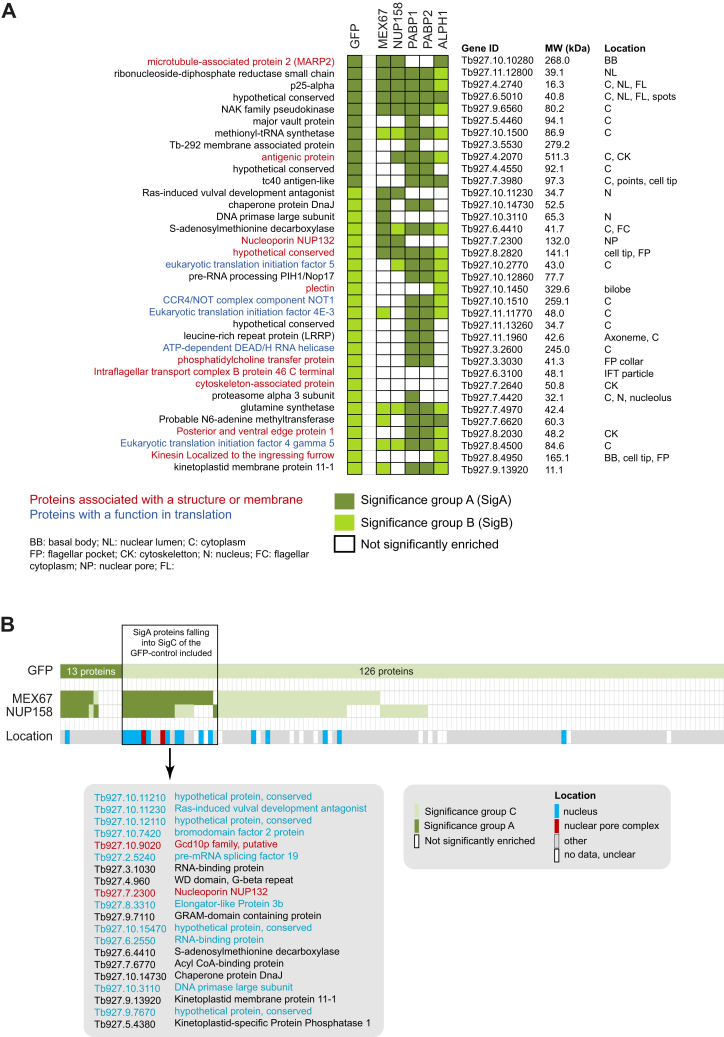
**Proteins biotinylated by the GFP-TurboID control.** A cell line expressing GFP-TurboID was used to detect background biotinylation. We have used different thresholds to define a protein as GFP positive for the different bait proteins (SigA, SigB, and SigC; details in the text). For further comparison, we have added respective TurboID data for the trypanosome mRNA decapping enzyme ALPH1 (63), which localizes to the cytoplasm and posterior pole granules. *A*, to control the BioID experiment of PABP1 and PABP2, proteins biotinylated by GFP-TurboID were defined using strict parameters (35 proteins, falling into SigA and SigB): only the proteins shown here were considered GFP positive. All GFP-positive proteins were removed from the list of proteins identified in the BioID with PABPs as baits, independent of the significance group. *B*, to control the BioID experiment of MEX67 and NUP158, we used less strict parameters to define the control (139 proteins, falling into SigA and SigC, were considered GFP positive). However, proteins that were identified with very high confidence with MEX67 and NUP158 BioID were included, if the GFP control was in SigC. Protein localization is mainly taken from TrypTag (20). PABP, poly(A)-binding protein; SigA, significant class A; SigB, significant class B; SigC, significant class C.

For the abundant cytoplasmic proteins PABP1 and PABP2, we chose strict parameters to define significance intervals for both the bait proteins and the GFP control (SigA: false discovery rate [FDR] = 0.01; s0 = 0.1; SigB: FDR = 0.05; s0 = 0.1). This defined 35 proteins as GFP positive, 13 in SigA and 24 in SigB (Table S1, *A* and *B*, Figs. 2 and 3*A*), which were removed from the list of PABP1 and PABP2 candidate interactors. The usage of less strict parameters for the GFP-control (SigC: FDR = 0.05; s0 = 2; as used for NUP158 or MEX67 mentioned later) would have resulted in the subtraction of *bona fide* PABP1/2 interactors, as for example, PAB1-binding protein PBP1 and even the bait PABP1 itself. Among the GFP-positive proteins were many involved in translation or associated with cytoskeleton or membranes (19, 20, 21, 22, 23, 24), as well as large proteins (26% > 100 kDa; whole genome: 10% > 100 kDa). Only two of this cohort were unique to the GFP BioID experiment, likely reflecting nonspecific interactions, whereas the remaining proteins were also identified in other BioID experiments (usually in more than one; nine in all five experiments).

We also applied SigA and SigB cutoffs to the less abundant proteins with a confined localization at the NPC, NUP158, and MEX67. However, we chose less strict parameters to define the GFP control (sigA: FDR = 0.01; s0 = 0.1, SigC: FDR = 0.05; s0 = 2) (Table S1, *A* and *C*, Figs. 2, and 3*B*). With these parameters, 139 proteins were defined as GFP positive (13 of these in SigA). About 50% of these 139 proteins were uniquely enriched in the GFP control. The others were removed from the list of NUP158- or MEX67-enriched proteins, except for proteins that were in SigA for the bait proteins and in SigC for GFP (listed in Fig. 3*B*). The latter cohort contained an enrichment in proteins with nuclear localization and even two proteins with localization to the NPC, including the *bona fide T. brucei* NPC component NUP132 (11, 25). In contrast, very few nuclear-localized proteins were among confirmed GFP-positive proteins (Fig. 3*B*).

In conclusion, we determined the appropriate filtering parameters to define the GFP control data for each individual BioID bait to exclude obvious false positives, while including known interaction partners. Notably, being GFP positive does not necessarily exclude the protein from being a true interacting protein; MARP2 (TbBBP268) was identified as GFP positive here but was identified by BioID with basal body protein TbPAC11 and TbBBP46 as baits. MARP2 localization to the basal body indicates that this is likely a true interactor (21). Likewise, some of the translation initiation factors and the RNA helicase within the cohort of GFP positives may be true PABP interactors, highlighting the need for careful consideration of the dataset.

### Cytoplasmic PABPs

**Figure 4:**
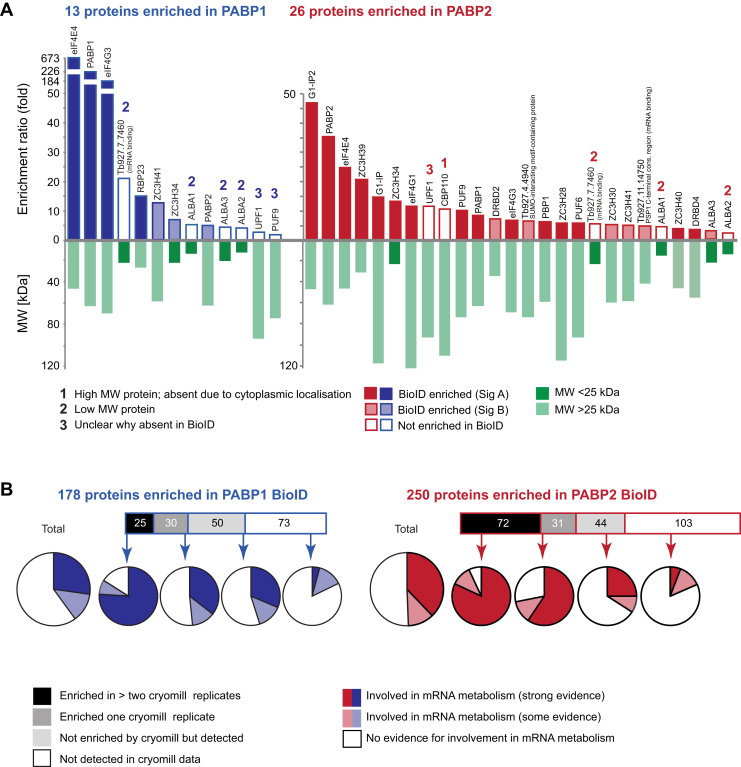
**Poly(A)-binding proteins (PABPs): comparing BioID and pulldown.***A*, all proteins significantly enriched in all four replicates of the PABP1 and PABP2 pulldown experiment are shown ranked according to their enrichment ratio (10). Proteins that are also identified in the respective BioID experiment and matching significance group A or B criteria are shown with *black* or *gray filling*, respectively. The molecular weight of all proteins is shown below, as we noticed a certain bias for loss of small proteins in BioID. Possible reasons of why a protein is absent from the BioID data are indicated. *B*, all proteins identified with the PABP BioID experiments in significance group A are listed, split into four groups, dependent on whether these proteins were found enriched in more than one of the pulldown experiments, enriched in only one of the pulldown experiments, detected in the pulldown experiments but not enriched or not detected. For each group of proteins, the fraction of proteins involved in mRNA metabolism is shown as a pie diagram (Table S2,*B* and *C*).

*T. brucei* has two cytoplasmic PABPs. Both PABP1 and PABP2 have cytoplasmic localizations under physiological conditions, but PABP2 localizes to stress granules when cells are starved (26, 27) and can also localize to the nucleus under certain conditions (26, 28). We have previously determined an interactome for PABP1 and PABP2 by cryomill affinity capture using GFP single-chain antibodies, from cells expressing C-terminal fusions of the PABPs to eYFP from endogenous loci (10). Data from four experiments (two with high and two with low salt conditions) were analyzed to yield a high confidence list of PABP PPIs that included only proteins that were at least twofold enriched in all four experiments, had no more than one zero-detection in the bait sample replicates and were no obvious contaminants. This resulted in a cohort of 13 proteins coimmunoprecipitated with PABP1, of which two proteins (eIF4E4 and eIF4G3) dominated with extremely high enrichment ratios of >150. PABP2 coimmunoprecipitated 26 proteins (Fig. 4*A*). All proteins coimmunoprecipitated with either PABP1 or PABP2 have either a known function in *T. brucei* mRNA metabolism, are known mRNA binders (Tb927.7.7460 and Tb927.11.14750 (29)) or have a localization reflecting RNA granules (Tb927.4.4940 (20)), suggesting a cohort of genuine PPIs. In contrast, the number of proteins identified with BioID was larger and included 178 (PABP1) and 250 (PABP2) proteins in SigA and 263 (PABP1) and 330 (PABP2) proteins in SigB (Table S2).

We first asked which of the affinity-purified proteins were also identified with BioID (Fig. 4*A*). Of 13 proteins identified in the PABP1 pulldown, four were in SigA of the BioID experiment, including the bait and the two highly enriched proteins eIF4E4 and eIF4G3. A further three proteins were in SigB. Six proteins were not enriched in the BioID experiment. Four of these are low molecular weight proteins (Fig. 4*A*) and may be missed because of their lower number of detectable unmodified peptides, which we recognized as a systematic problem of BioID (discussed in Conclusion section). The remaining two proteins, PUF9 and UPF1, were the least enriched proteins in the PABP1 pulldown and hence may suggest a less stable or physically more distant and indirect association with PABP1. Of the 26 proteins identified in the PABP2 pulldown, 15 are also in BioID SigA, including the bait and six proteins with highest enrichment ratios in the pulldown. A further six proteins were in the SigB cohort. Of the remaining five, three are low molecular weight and may not be detected as suggested previously. The fourth is a large nuclear protein (CBP110) that possibly remains in the cytoskeleton fraction removed prior to incubation with the streptavidin beads. The fifth protein is, again, UPF1.

We next asked how many proteins identified by BioID were also present in the pulldown datasets (Fig. 4*B*). Of the 178 proteins identified in the PABP1 BioID (SigA), 25 were present in at least two replicates of the pulldown, 30 were enriched in one replicate, 50 were not enriched but detected, and 73 were not detected. Of the 250 proteins from the PABP2 BioID (SigA), we detected 72 in at least two of the replicates of the pulldown, a further 31 in one replicate, 44 not enriched but detected, and 103 proteins undetected. PABP-interacting proteins are expected to be mainly involved in mRNA metabolism. To evaluate these data further, we manually assessed evidence for involvement in mRNA metabolism for each protein, based on conserved domains, known homologs, and published data. About 40 and 49% of PPIs identified by BioID in confidence group SigA for PABP1 and PABP2, respectively, had evidence for a role in mRNA metabolism. As expected, the number of proteins with a role in mRNA metabolism was highest (>75%) among the BioID proteins that were also identified by affinity capture and lowest (<25%) among the BioID proteins undetected by affinity isolation.

While there is overlap between BioID and pulldown PPI datasets, BioID identifies a significantly larger number of potential interactors, partly explained by the complexity of PABP interactions and the dynamic nature of ribonucleoprotein complexes in which the PABP function. Consistent with this, a recent study (30) applied BioID to elucidate the PPIs of two *T. brucei* RNA-binding proteins, RBP9 and RBP10, uncovering a similar number (>200) of high-confidence interactors.

BioID appears to have a false-positive rate among the high-confidence SigA cohort of about 50%. This is likely because of the less-confined (cytoplasmic) localization of PABP1 and PABP2 and perhaps in addition by their function: PABPs are mobile and interact with many complexes that themselves may have peripheral proteins not obviously involved in mRNA metabolism. Moreover, PABPs are involved in translation and thus in close proximity to many nascent proteins that they potentially biotinylate despite the absence of direct interaction. Note, however, that ribosomal proteins were not found biotinylated, excluding random biotinylation of the entire polysomal complex.

### MEX67

MEX67 forms a heterodimer with Mtr2, and this complex is the major mRNA export complex and is conserved across most eukaryotes (31, 32). MEX67 binds its mRNA targets both directly or indirectly *via* adaptor proteins and mediates mRNA export to the cytoplasm by interacting with FG-type nucleoporins. While mRNA export in trypanosomes differs in several aspects from the pathway described in opisthokonts (33), TbMEX67 (Tb927.11.2370) has been well studied in the past and appears to have a conserved function in mRNA export (17, 33, 34, 35). Two datasets from pulldowns are available for *T. brucei* MEX67. A classical immunoprecipitation with a C-terminal PTP (Protein C- tobacco etch virus protease cleavage site - Protein A) tag identified Mtr2 and importin 1, a transport receptor for nuclear import (35), whereas a cryomill affinity capture using a C-terminally GFP-tagged MEX67 as bait identified many nucleoporins (11). We reanalyzed the latter affinity capture data (11) for three different extraction conditions to generate a list of enriched proteins (Table S4*B*). We have then performed BioID with MEX67 to compare the PPIs identified by these different methods.

**Figure 5:**
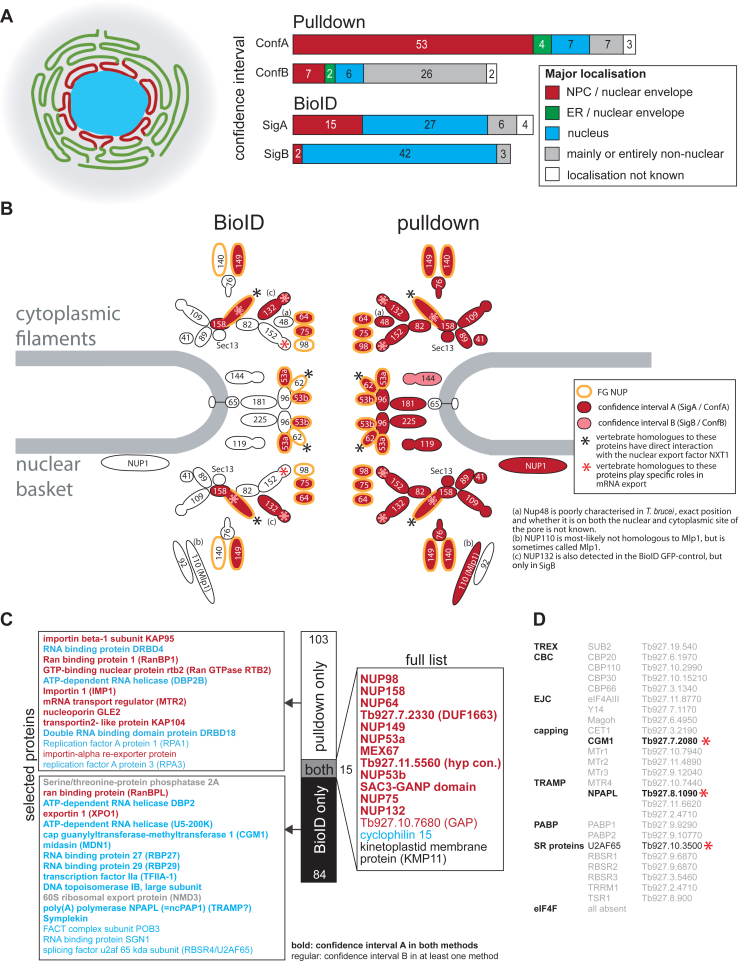
**MEX67: comparing BioID and pulldown.***A*, proteins identified in the MEX67 pulldown and BioID experiments are grouped according to their cellular localizations based on TrypTag (20). A corresponding color-code scheme is shown on the *left*. *B*, schematic representation of the trypanosome NPC (11). Proteins identified in the BioID or pulldown experiments are filled in *red* or *light red* for significance group A or B, respectively. *C*, comparison between proteins identified in the pulldown and the BioID. All proteins identified with both methods are listed as well as selected proteins for proteins exclusively identified in the BioID or pulldown. *D*, list of proteins involved in selected nuclear mRNA maturation processes (33) and in translation (eIF4F and PABPs). Only three of these proteins were identified in the BioID experiment (*red asterisk*) in significance group A (*black*, *bold*) or B (*black*, *regular*). Full details on the MEX67 BioID experiment are listed in Table S3*B*. A corresponding color-code scheme is shown on the *left*. CBC, cap binding complex; EJC, exon junction complex; NPC, nuclear pore complex; PABP, poly(A)-binding protein; TRAMP, Trf4–Air2–Mtr4, polyadenylation; TREX, couples transcription and export.

In contrast to the PABP1 and PABP2 datasets, the number of MEX67 PPIs identified with BioID (99) was similar to the number of PPIs identified by pulldown (118 proteins) (Fig. 5*A*). We suggest that this is likely a consequence of the more confined localization of the MEX67 protein, which results in less bystander labeling.

MEX67 BioID identified only nine proteins with a non-nuclear localization, spread over both SigA and SigB cohorts, and the majority of proteins were localized to the nucleoplasm (69 proteins) or the NPC (17 proteins) (Fig. 5*A*). SigA contained 15 core nucleoporins, whereas SigB showed enrichment of proteins localized to the nucleoplasm. Proteins with nuclear localization only identified by BioID could be proteins that interact with nuclear proteins only transiently, preventing their detection in the pulldown experiment.

Pulldown-identified PPIs differ from the corresponding BioID PPIs in containing a larger number of nucleoporins and a smaller number of nucleoplasmic proteins, and, in particular, for confidence group B, a higher number of non-nuclear proteins. Also specific to the pulldown PPIs were proteins localized to the endoplasmic reticulum (ER) and nuclear envelope: these likely coprecipitated with the NPCs but are not in close contact with MEX67 and hence not sampled with BioID (Fig. 5*A*). However, it should be considered that any transmembrane, vesicular, or intralumenal (perinuclear cisterna) proteins might also be inaccessible to the BioID labeling but not to direct physical connection–mediated affinity capture.

While MEX67 coprecipitated essentially the entire NPC, MEX67 BioID selectively identified six of nine FG-repeat proteins exposed to central channel (Fig. 5*B*). The only non-FG-repeat NUP identified by BioID is NUP132, which is also enriched with the GFP-bait control (SigB) and may thus have higher exposure to random biotinylation. Thus, for MEX67, BioID captured only proteins directly interacting with MEX67, whereas the pulldown identified the entire NPC, likely driven by high-affinity interactions between individual nucleoporins.

Similar to PABPs, there was little concordance between the PPIs identified by the two different methods, and only 15 proteins of 202 were common (Fig. 5*C* and Table S3*B*). Additional to MEX67, there were 12 proteins with localization to the NPC or nuclear envelope, namely (i) eight nucleoporins (compare Fig. 5*B*), (ii) Tb927.6.890, unique to kinetoplastida, but possessing a SAC3/GANP/THP3 domain at its N terminus; Sac3 from yeast is a well-known interactor of MEX67-Mtr2 that localizes to the cytoplasmic side of the NPC (36), (iii) Tb927.7.2330 and Tb927.11.5560, two proteins of unknown function, and finally (iv) Tb927.10.7680, a GTPase-activating protein (RabGAP-TBC domain, TBC-RootA (37)); TBC-RootA is likely involved in the GTP-dependent mRNA transport *via* the small GTPase Ran (11). Ran, Ran-binding protein, and MEX67b (38), all high-confidence interactors in the pulldown, were also enriched in the BioID PPI dataset but below a significance cutoff. Two further proteins common to both BioID and pulldown are cyclophilin (localization to the nucleolus, unknown function) and KMP11 (a protein of the basal body and flagellum attachment zone) (20, 39), both likely contaminants. Surprisingly, the major MEX67 interactor, Mtr2, was only identified by pulldown, likely because of the low molecular weight of Mtr2 (15.8 kDa).

About 103 proteins were unique to the pulldown, 61 of these in confidence group A. Most (40 proteins) of these localized to the NPC or nuclear envelope and included most nucleoporins (compare Fig. 5*B*), (putative) transport proteins including Ran-binding protein 1, importin 1, importin beta, Ran RTB2, and many hypothetical proteins. Four proteins localized to the nuclear envelope and ER. Seven had nuclear localizations, including the RBPs DRBD4, DBP2B, and DRBD18, whereas 10 had non-nuclear or unknown localizations.

In contrast, of 84 proteins unique to the BioID dataset, 68 had a nuclear localization, four with NPC and only 12 had non-nuclear or unknown localization. NPC-associated proteins unique to the BioID were exportin-1 (XPO1), NMD3, and Ran-binding protein RanBPL. Interestingly, NMD3 is implicated in nuclear export of mRNA of procyclin-associated genes, a process shown to be disrupted by silencing of NMD3, XPO1, or MEX67 (40). This finding extended the prototypic function of NMD3 and XPO1 in rRNA export and is suggestive of overlapping and interdependent nuclear export routes for mRNA and rRNA, for which there is also evidence in yeast (41). While the absence of NMD3 and XPO1 in the pulldown argues against a stable interaction with MEX67, their high enrichment in BioID datasets suggests transient interactions with MEX67.

Among the nuclear-localized proteins were many RBPs and proteins with functions in nuclear mRNA processing, including spliceosomal factors (U5-200K, RBSR4/U2AF65, splicing factor 3B, U4/U6 small nuclear ribonucleoprotein PRP3), an exosome subunit RRP6, the noncoding poly(A) polymerase NPAPL, the cap guanylyltransferase-methyltransferase 1 (CGM1), and proteins involved in transcription. However, the majority of proteins known to be involved in nuclear mRNA metabolism (33) was not identified with either method (Fig. 5*D*).

To summarize, both BioID and pulldown detected the expected interactions of MEX67 with FG-type nucleoporins. In fact, these are likely the major interactions; as in yeast, MEX67 can be fused to such a nucleoporin and still fulfill its essential functions (42). Apart from these FG-type nucleoporins, there was little coincidence between the PPIs identified by the two methods. While the pulldown mainly coimmunoprecipitated the entire NPC, BioID identified nuclear proteins involved in mRNA metabolism and many RBPs, potentially reflecting transient interactions between MEX67 and its mRNA substrates that are missed in the pulldown.

Altogether, BioID is a valuable tool for interrogation of MEX67 function as it clearly discriminates components of the NPC engaging in direct interaction, as the subset NUPs exposed the inner pore channel. On the other hand, the pulldown detects many indirect interactions because of high stability of NPC subunit interactions. The latter provides equally meaningful information, as MEX67 indeed, is considered to exhibit some of the characteristics of a mobile nucleoporin, because of its significant presence at the NPC (42). Furthermore, BioID appears to identify transient interactions, as indicated by the enrichment of proteins with a function in mRNA binding and nuclear localization, albeit requiring validation.

### The nucleoporin NUP158

**Figure 6:**
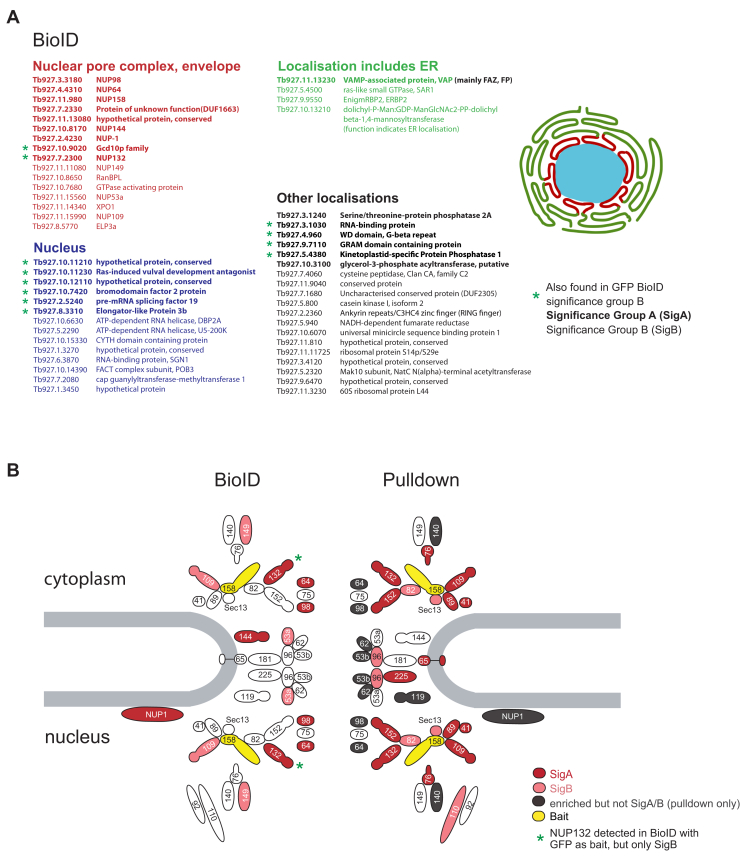
**BioID with NUP158.***A*, all proteins identified in the NUP158 BioID are listed, grouped according to their intracellular localizations based on TrypTag (20). *B*, schematic representation of the trypanosome NPC (11). Proteins identified in the NUP158 BioID or pulldown experiments are filled in *red* or *light red* for significance intervals A or B, respectively. The bait protein NUP158 is filled in *yellow*. Proteins enriched in the pulldown falling outside the SigA/B cutoff are filled in *gray*. NPC, nuclear pore complex. SigA, significant class A; SigB, significant class B.

NUP158 is an FG-NUP/alpha-solenoid and ortholog of outer ring nucleoporins ScNup145 and HsNup98-96. Cryomill affinity capture was repeated as described (11) but subjecting the entire elution to triplicate LC–MS/MS analysis. We detected coprecipitated 23 nucleoporins, eight falling into SigA and five into SigB (Figs. 6*B*, S1 and Table S3). Among these are NUP109, Sec13, NUP41, NUP82, NUP89, NUP132, and NUP152; together with NUP158, these eight proteins form the outer ring complex (11). Further significant interactors are the nuclear basket protein NUP110, NUP76, NUP48 and inner ring proteins NUP65, NUP96, and NUP225. Many additional NPC-associated proteins such as NUP140, the inner ring proteins NUP62, NUP53b, and NUP119, the FG NUPs NUP64 and NUP98, and the lamina protein NUP-1 were also enriched but fall outside the SigA/B cutoff. About 18 additional proteins were identified as significant interactors (Table S3), of which five localize to the NPC and further five to the nucleus (20).

NUP158 BioID identified 53 proteins (Fig. 6*A* and Table S3): 10 proteins in SigA (including five nucleoporins), a further 12 in SigA that also appear in the GFP-control SigC detections, and 31 proteins in SigB. In comparison to the PABP and MEX67 BioID experiments, this is the shortest list of biotinylated proteins, likely reflecting the highly confined localization of NUP158. Of these 53 proteins, 13 localized to the NPC, 16 to the nucleus, and five to the ER/nuclear envelope (20).

Concordance between BioID and pulldown PPIs was once again poor (Fig. 6*B*). Many nucleoporins identified as interaction partners in the pulldown were not identified in the BioID (NUP140, NUP76, NUP41, NUP89, SEC13, NUP82, NUP152, and NUP110) and, vice versa, others were unique to the BioID (NUP144, NUP149, and NUP53a) or outside the pulldown SigA/B cutoff (NUP98 and NUP64). The only nucleoporins in common were FG-NUPs NUP109 and NUP13, and there was just one additional interactor Tb927.11.13080 shared between pulldown and BioID datasets (Table S3). This latter is a protein of unknown function but localizes to the NPC (20)). Hence, both methods identify meaningful, but significantly different, PPIs for NUP158. The pulldown identifies adjacent outer ring proteins that are partly absent in BioID. A potential reason for the absence could be that NUP158, located within the rigid structure forming the outer rings of the NPC, has a limited labeling radius because of immobility, steric constraints, or even the local biochemical environment. Therefore, although pulldown is superior identification of PPIs for a strictly localized nucleoporin, additional valuable data can be obtained with BioID.

## Conclusions

Collectively, we found surprisingly little concordance between proteins identified by BioID and by affinity capture. This strongly suggests that these methods should be viewed as providing a distinct PPI for a given bait rather than offer equivalents or alternatives. A similar comparative interactomics study to the one here has been performed for several chromatin-associated protein complexes in human cell lines (43, 44) with broadly similar outcome; BioID generally produced larger interactomes, and the concordance between pulldown and BioID was poor, *albeit* that both techniques identified meaningful PPIs. Our dataset however analyzed a cohort of bait proteins with considerably greater variation of location and positional constraints to provide additional insight.

For soluble and unconstrained cytoplasmic proteins with a high potential for nonspecific interactions, BioID has a significant level of bystander labeling. By contrast, BioID with MEX67, a more restricted bait, identified exclusively FG-NUPs of the inner pore channel, whereas pulldown coprecipitated the entire NPC. Thus, the optimal method depends on the localization of the protein of interest. Ideally, both methods are used in parallel, and recently, a hybrid tag was introduced combining both biotin ligase and an epitope tag for pulldown (45). Several variants of BioID aiming to overcome some of the pitfalls of BioID are available and include an inducible system with target-specific biotinylation only occurring when the biotin ligase is attached to the bait using a dimerizing agent (46) and a split BioID, where biotinylation can only occur once two proteins carrying partial BioID tags interact (conditional biotinylation) (47).

A major weakness of BioID beyond bystander identifications is that many low-molecular weight proteins are unidentified. Most strikingly, here was the absence of Mtr2 from the MEX67 BioID data. In general, small proteins are harder to detect by MS than large proteins as there are simply less peptides to detect, but this applies to both pulldown and BioID. However, first, BioID relies on surface-exposed lysine residues, and probability of occurrence depends on protein size amongst other parameters. Second, in pulldown analysis, peptides of the entire protein are potentially available for MS, as proteins are fully, or near fully, eluted from the beads, but in BioID, peptides are eluted from beads by trypsin digestion, and any biotinylated peptides remain attached to the beads (Table S4). For low-molecular weight proteins, the likelihood that lysine residues are inaccessible is lower than for larger proteins, as small proteins are less likely to “bury” lysine residues in their core. This can explain the systematic absence of expected interactors with a low molecular weight from BioID but not from the pulldown. One potential way to increase elution of biotinylated peptides would be to add excess biotin before the proteolytic digest to saturate all biotin-binding sites and hence restrict rebinding of biotinylated peptides to the beads after their release by the protease. There is also an alternative approach for purification: trypsination of cell lysates prior to purification followed by purification of biotinylated peptides using a biotin-antibody rather than streptavidin has been shown to result in a higher recovery rate of biotinylated peptides (48, 49). However, this approach comes at the cost of increased background from nonspecific binding, excluding the use of stringent washes as facilitated by the strong biotin–streptavidin interaction.

Altogether, this study offers guidance for choosing the most appropriate method for protein complex characterization, dependent on localization and positional constraints. Furthermore, it highlights potential pitfalls concerning experimental design and data interpretation. Overall, BioID and affinity capture are complementary with each approach elucidating a unique subset of PPIs for a given target protein. Hence, combination of both methods can be leveraged for more complete interactome mapping.

## Experimental procedures

### Cell culture and genetic manipulation

*T. brucei* Lister 427 procyclic cells were cultured in SDM-79 (50). The generation of transgenic trypanosomes was done using standard methods (51). All fusion proteins were expressed from their endogenous locus as described (52), modified to result in fusing TurboID and one HA tag to the C terminus of the protein. eYFP was expressed fused to TurboID-HA using an inducible expression system based on tetracycline (52).

### Western blot

Western blotting was performed using standard methods. Detection of biotinylated proteins was done with Streptavidin-IRDye680LT (LI-COR) (1:10,000 dilution). Binding immunoglobulin protein (BiP) was detected using anti-BiP (1:200,000 dilution) (kind gift of James D. Bangs, University at Buffalo).

### Immunofluorescence

About 1 × 10^7^ cells at 5 × 10^6^ cells/ml were washed once in 1 ml SDM79 without hemin and serum and resuspended in 500 μl PBS. For fixation, 500 μl of 8% paraformaldehyde was added for 20 min while rotating. About 7 ml PBS with 20 mM glycine were added, cells were pelleted, resuspended in 150 μl PBS, and spread on polylysine-coated slides (in *circles* drawn with a hydrophobic pen). After 15 min, cells had settled to the slide, surplus PBS was removed, and cells were permeabilized with 0.5% Triton X-100 in PBS. Slides ware rinsed in PBS, and cells were then blocked in 3% bovine serum albumin (BSA) in PBS for 30 min, followed by 60 min incubation with rabbit-mAb-anti-HA C29F4 (1:500 dilution; Cell Signaling Technology) and with Streptavidin-Cy3 (1:200 dilution; Jackson Laboratories) in PBS/3% BSA. Slides were washed in PBS (three times for 5 min) and incubated with anti-rabbit Alexa Fluor Plus 488 (1:500 diluiton) in PBS/3% BSA for 1 h; the last 10 min were done in the presence of 4′,6-diamidino-2-phenylindole (DAPI) (0.1 μg/ml). Slides were washed 3 × 5 min in PBS and embedded into ProLong Diamond Antifade Mountant (Thermo Fisher Scientific). Images were recorded as Z-stacks (100 images with 100 nm distance) on a custom build TILLPhotonics iMic microscope equipped with a Sensicam camera (PCO AG, 6.45 m/pixel) und 100× oil immersion (numerical aperture = 1.4) objectives (Olympus). Filter sets were (i) excitation (ex): 320 to 380 nm, dichroic (dc): 400 to 430 nm, and emission (em): 438 to 486 nm (DAPI), (ii) ex: 430 to 474 nm, dc: 585 nm, and em: 489 to 531 nm (Alexa Fluor Plus 488) and (iii) ex: 540 to 580 nm, dc: 585 nm, and em: 592 to 664 nm (Cy3). For each image, the exposure times were 50 ms for DAPI and 500 ms for all other fluorophores. Images were deconvolved using Huygens Essential software (Scientific Volume Imaging BV) and are either presented as a single plane or as a Z-stack projection (sum slices), as indicated.

### Affinity enrichment of biotinylated proteins and on-bead tryptic digests

Cells were maintained at a density of 1 × 10^6^ to 10^7^ cells per ml and harvested at a cell density of 5 × 10^6^ cells per ml. The eYFP control was induced by addition of tetracycline at a concentration of 1 μg/ml 24 h prior to harvesting. No extra biotin was added for induction of labeling, as we found the biotin concentration in the SDM79 medium (827 nM) to be sufficient for high-level biotinylation.

About 5 × 10^8^ cells were used in each replicate. Cells were harvested at 1400*g*, washed once with serum-free medium, and pellets were rapidly frozen in liquid nitrogen and stored at −80 °C. For isolation of biotinylated proteins, each cell pellet was resuspended in 1 ml lysis buffer (0.5% octylphenoxypolyethoxyethanol, 0.1 M Pipes-NaOH (pH 6.9), 2 mM ethylene glycol-bis(β-aminoethyl ether)-*N*,*N*,*N*′,*N*′-tetraacetic acid, 1 mM MgSO_4_, 0.1 mM EDTA, complete EDTA-free protease inhibitor cocktail [Roche]) and incubated for 15 min at room temperature (RT) in an orbital mixer. Soluble and nonsoluble fractions were separated by centrifugation (14,000*g*, 5 min, 4 °C) and the soluble fraction was incubated with 100 μl streptavidin-linked Dynabeads (MyOne Streptavidin C1; Thermo Fisher Scientific) for 1 h at 4 °C under gentle mixing. Beads were washed twice in 1 ml buffer 1 (2% [w/v] SDS in water) once in 1 ml buffer 2 (0.1% [w/v] deoxycholate, 1% Triton X-100, 1 mM EDTA, 50 mM Hepes [pH 7.5], and 500 mM NaCl), once in 1 ml buffer 3 (250 mM LiCl, 0.5% octylphenoxypolyethoxyethanol, 0.5% [w/v] deoxycholate, 1 mM EDTA, 10 mM Tris–HCl [pH 8.1]), and once in 1 ml buffer 4 (50 mM Tris–HCl [pH 7.4], 50 mM NaCl); each washing step was 8 min at RT under orbital shaking. Beads were then prepared for tryptic digestion by washing three times in 500 μl ice-cold 50 mM NH_4_HCO_3_, resuspension in 40 μl of the same buffer supplemented with 10 mM dithiothreitol, and incubation in a thermomixer at RT for 1 h. Iodoacetamide was added to a concentration of 20 mM, followed by incubation in the dark at RT for 30 min. Finally, 5 μg/ml proteomics-grade trypsin (SOLu-Trypsin; Sigma–Aldrich) was added to the beads. The digest was done overnight at 30 °C in a thermomixer (1000 rpm). After removal of the first eluate, beads were resuspended in 50 μl 50 mM NH_4_HCO_3_ supplemented with 10 mM dithiothreitol and 5 μg/ml MS-grade trypsin and incubated in a thermomixer at 37 °C for 1 h. The eluate was combined with the first eluate, and both were lyophilized in a Speed-vac (Christ alpha 2–4). LoBind tubes (Eppendorf) were used throughout.

### MS and proteomics analysis

BioID-eluted peptides were resuspended in 50 mM NH_4_HCO_3_ and passed over C_18_ stage tip columns as described (53) and then analyzed by LC–MS/MS on an Ultimate3000 nano rapid separation LC system (Dionex) coupled to an LTQ Q-exactive mass spectrometer (Thermo Fisher Scientific). Resulting spectra were processed using the intensity-based label-free quantification (LFQ) in MaxQuant, version 1.6.16 (Max Planck Institute of Biochemistry, Martinsried) (54, 55). Minimum peptide length was set at six amino acids allowing a maximum of two missed cleavages, and FDRs of 0.01 were calculated at the levels of peptides, proteins, and modification sites based on the number of hits against the reversed sequence database. Data from TurboID were in addition searched for lysine biotinylated peptides (+226.078 Da). LFQ data were analyzed using Perseus (18). For statistical analysis, LFQ values were log_2_ transformed, and missing values were imputed from a normal distribution of intensities around the detection limit of the mass spectrometer. These values were subjected to a Student’s *t* test comparing an untagged control (*wt* cells) triplicate sample group to the TurboID-tagged protein triplicate sample groups, including the GFP control. −log_10_ *t* test *p* values were plotted *versus t* test difference to generate multiple volcano plots (Hawaii plot, Fig. 2). Potential interactors were classified according to their position in the Hawaii plot, applying cutoff curves for “significant class A” (SigA; FDR = 0.01, s0 = 0.1), “significant class B” (SigB; FDR = 0.05, s0 = 0.1), and “significant class C” (SigC; FDR = 0.05, s0 = 2), respectively. The cutoff is based on the FDR and the artificial factor s0, controlling the relative importance of the *t* test *p* value and difference between means (at s0 = 0 only the *p* value matters, whereas at nonzero s0, the difference of means contributes).

### Cryomill affinity capture

For affinity capture of PABP1, PABP2, and MEX67, we used data from our previous studies (10, 11). MEX67 data were reanalyzed for three different extraction conditions (higher stringency buffer 1: 20 mM Hepes, pH 7.4, 250 mM NaCl, 0.5% Triton, plus protease inhibitors, and two low stringency stabilizing buffers: buffer 2A—20 mM Hepes, pH 7.4, 250 mM sodium citrate, 0.5% Tween-20, plus protease inhibitors; buffer 2B—20 mM Hepes, pH 7.4, 20 mM NaCl, 50 mM sodium citrate, 0.5% Tween-20, plus protease inhibitors) and compared with a negative control, omitting the GFP tag. Potential interactors were assigned to two confidence intervals termed confidence A (confidence group A) for proteins enriched greater than twofold under all three conditions and confidence B for proteins enriched greater than twofold in at least one condition. The NUP158 cryomill affinity enrichment purification analysis was repeated essentially as described (11) but subjecting the entire elution to triplicate LC–MS/MS analysis. In brief, NUP158 endogenously tagged with eYFP at the C terminus was extracted in buffer 3 (20 mM Hepes [pH 7.4], 250 mM NaCl, 0.5% CHAPS, complete EDTA-free protease inhibitor cocktail), captured on magnetic anti-GFP nanobody beads (GFP-Trap Magnetic Agarose; Chromotek) and washed four times with buffer 3. Proteins were eluted by on-bead tryptic digest and analyzed by LC–MS/MS on an Ultimate3000 nano rapid separation LC system (Dionex) coupled to an Orbitrap Fusion mass spectrometer (Thermo Fisher Scientific). Data were analyzed as described (56) and detailed previously.

## Data availability

All proteomics data have been deposited at the ProteomeXchange Consortium *via* the PRIDE partner repository (57) with the dataset identifier PXD031245. The proteomics data for the PABP1 and PABP2 pulldown have the dataset identifier PXD008839.

## Supporting information

This article contains supporting information(19, 20, 21, 22, 23, 24, 25, 58, 59, 60, 61, 62).

## Conflict of interest

The authors declare that they have no conflicts of interest with the contents of this article.
