# Supplementary figures and images for: Impact of inherent biases built into proteomic techniques: Proximity labeling and affinity capture compared
Source: J Biol Chem. 2022 Nov 19;299(1):102726. doi: 10.1016/j.jbc.2022.102726 (PMC9791439; doi:10.1016/j.jbc.2022.102726)

FIGURE S1

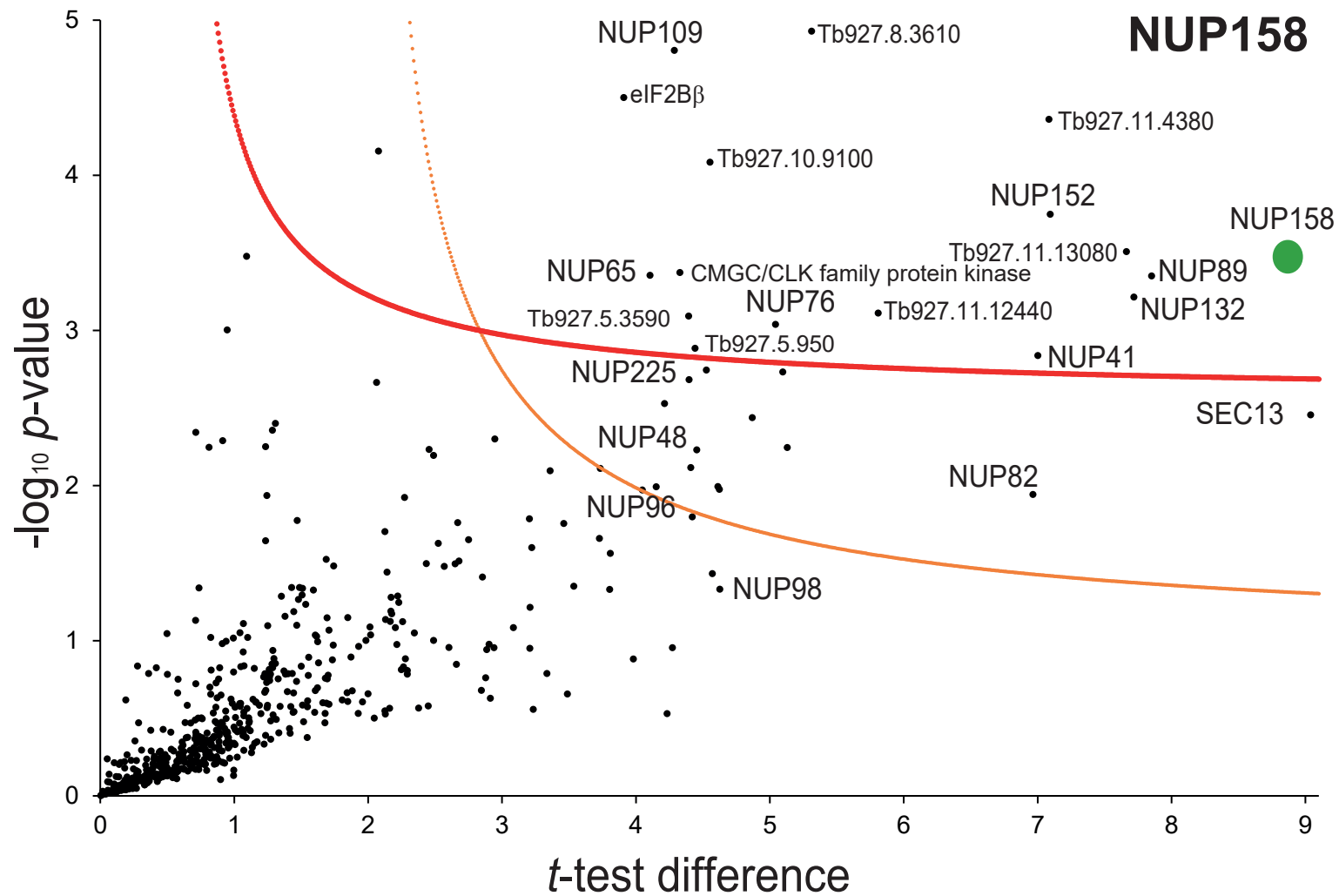

Supplement: Figure S1 [file mmc5.pdf]
